# Insights into the Binding of Dietary Phenolic Compounds to Human Serum Albumin and Food-Drug Interactions

**DOI:** 10.3390/pharmaceutics12111123

**Published:** 2020-11-21

**Authors:** Anallely López-Yerena, Maria Perez, Anna Vallverdú-Queralt, Elvira Escribano-Ferrer

**Affiliations:** 1 Department of Nutrition, Food Science and Gastronomy XaRTA, Faculty of Pharmacy and Food Sciences, Institute of Nutrition and Food Safety (INSA-UB), University of Barcelona, 08028 Barcelona, Spain; naye.yerena@gmail.com (A.L.-Y.); mariaperez@ub.edu (M.P.); avallverdu@ub.edu (A.V.-Q.); 2 Laboratory of Organic Chemistry, Faculty of Pharmacy and Food Sciences, University of Barcelona, 08028 Barcelona, Spain; 3 CIBER Physiopathology of Obesity and Nutrition (CIBEROBN), Institute of Health Carlos III, 28029 Madrid, Spain; 4 Pharmaceutical Nanotechnology Group I+D+I Associated Unit to CSIC, Biopharmaceutics and Pharmacokinetics Unit, Department of Pharmacy and Pharmaceutical Technology and Physical Chemistry, Institute of Nanoscience and Nanotechnology (IN2UB), Pharmacy and Food Sciences School, University of Barcelona, 08028 Barcelona, Spain

**Keywords:** plasma protein binding, distribution, bioavailability, molecular property, noncovalent interaction, pharmacokinetics

## Abstract

The distribution of drugs and dietary phenolic compounds in the systemic circulation de-pends on, among other factors, unspecific/specific reversible binding to plasma proteins such as human serum albumin (HSA). Phenolic substances, present in plant-derived feeds, foods, beverages, herbal medicines, and dietary supplements, are of great interest due to their biological activity. Recently, considerable research has been directed at the formation of phenol–HSA complexes, focusing above all on structure–affinity relationships. The nucleophilicity and planarity of molecules can be altered by the number and position of hydroxyl groups on the aromatic ring and by hydrogenation. Binding affinities towards HSA may also differ between phenolic compounds in their native form and conjugates derived from phase II reactions. On the other hand, food–drug interactions may increase the concentration of free drugs in the blood, affecting their transport and/or disposition and in some cases provoking adverse or toxic effects. This is caused mainly by a decrease in drug binding affinities for HSA in the presence of flavonoids. Accordingly, to avoid the side effects arising from changes in plasma protein binding, the intake of flavonoid-rich food and beverages should be taken into consideration when treating certain pathologies.

## 1. Introduction

The biological activities of dietary phenolic substances, present in plant-derived feeds, foods, beverages, herbal medicines, and dietary supplements, are of great interest. Polyphenols, which can be classified as flavonoids and non-flavonoids, contain, in addition to other substituents, at least one aromatic ring with one or more hydroxyl groups [1,2]. Flavonoids are a group of natural substances with variable phenolic structures as flavonols, flavan-3-ols (monomeric and polymeric structures), flavones, isoflavones, flavanones, and anthocyanidins. On the other hand, stilbenes, hydrolyzable tannins, lignans, and phenolic acids can be classified as non-flavonoids [3].

The absorption, distribution and elimination of dietary phytochemicals depend on their intestinal permeability and the influence of pre-systemic enzymes and/or transporters [4]. As systemic exposure can reflect tissue exposure, greater bioavailability should result in higher levels in tissues. Bioavailability is defined as the rate and the extent to which the active ingredient/phytochemical or moiety is absorbed from the ingested matrix and becomes available at the site of action [5]. It is well known that phenolic compounds have a low oral bioavailability, and undergo an extensive biotransformation mediated by phase I and phase II reactions in enterocytes and the liver, as well as by gut microbiota [6]. Polyphenol metabolites are also attracting research interest as their biological effects can be similar to or greater than those of the parent compounds [6,7]. Paradoxically, despite low oral bioavailability, most of the phenolic compounds have proven to have significant biological effects [6].

Once a xenobiotic has entered the systemic circulation, its rate of distribution to the various tissues of the body will be influenced by tissue hemodynamics (blood flow) and the ease with which it can cross the lipoidal cell membranes, either by passive diffusion or by passive/active facilitated transport (carrier-mediated) [8]. Nevertheless, the extent of distribution depends on partitioning into fat and other tissues and on unspecific/specific reversible binding to plasma proteins [9]. Plasma proteins, also called serum proteins, constitute important organic components consisting of simple as well as conjugated proteins [10]. Drugs are transported in the circulation either in a free form, dissolved in the aqueous phase of plasma, or in complex bonds with plasma proteins [11] in varying degrees [12]. Following the principle of reversible equilibrium and the law of mass action [13], an equilibrium exists between bound and free (unbound) molecular forms—additionally because binding is generally reversible [12]. Only the free form is capable of diffusing through membranes and from the vascular space into tissues, being eliminated by metabolism or excretion [14], and therefore pharmacological activity is exerted by the free drug concentration [15,16]. The fraction of a xenobiotic bound to a plasma protein depends on protein affinity towards the compound, protein and compound molar concentration, as well as on the possible competition with other endogenous and exogenous compounds for binding sites [17]. Generally, acidic compounds tend to bind to albumin, basic compounds to α_1_-acid glycoprotein (AAG), neutral compounds can be bound to both human serum albumin (HSA) and AAG, and neutral lipophilic compounds to lipoproteins. Other proteins, such as α-globulin, transcortin, fibrinogen, sex-hormone-binding globulin, and thyroid-binding globulin, bind specific compounds [18,19].

The free drug/xenobiotic concentration depends on the unbound drug clearance and dose, and is not usually changed by plasma protein binding (PPB) [16]. At a steady state, the free drug concentration remains balanced on both sides of any biomembrane [16]. As drug clearance occurs, a new equilibrium between bound and unbound forms is reached, which acts to maintain the free drug fraction [14]. Drug–protein complexes in plasma also serve as a drug reservoir, replacing what is removed by various distribution and elimination processes [8,15,17,20].

The free drug fraction is the ratio between the free drug concentration and total drug plasma concentration, which has values between zero (totally bound) and one (totally free), and remains constant in normal physiological conditions and at low molar drug concentrations. Only if the free fraction remains constant can the total plasma concentration be considered a good measure of changes in the unbound drug/xenobiotic concentration. This concept is important because the total plasma concentration is what is usually measured and not the unbound concentration, which is only occasionally determined. Although at the therapeutic concentration of most drugs, the molar concentration of unbound drugs is usually low in comparison with the molar concentration of the protein binding sites [13,21], in some pathophysiological conditions, the free drug fraction can be reduced/increased with ensuing changes in the distribution process, either by an altered protein–drug affinity or by a change in plasma protein levels [16].

Although it is traditionally considered that only the free form is capable of diffusing through membranes, recent studies have hypothesized that HSA facilitates Dp44mT delivery to the lysosomes by internalization through a carrier mediated transport mechanism enhancing its anti-cancer activity [22]. In addition, an in vitro study has shown for proteins with a net negative charge such as albumin, and for drugs highly bound to albumin and in the physiological albumin concentration range, a protein-mediated uptake mechanism [23]. It was observed in hepatocytes and cardiac myocytes. Certainly, this requires further intense investigation since PPB is an important process that determines the pharmacological activity and pharmacokinetics of drugs and other xenobiotics [12,24], and the impact of PPB on the efficacy and safety of a treatment needs to be better understood [25].

As with other xenobiotics, the distribution of phenolic compounds to the various tissues of the body is influenced by unspecific, reversible binding events to plasma protein [9]. PPB and phenolics have been the subject of numerous recent studies, which have focused above all on structure–affinity relationships. The aim of this review is to summarize how structural modifications affect the affinity of the main dietary polyphenols and their metabolites for HSA and to elucidate the main factors involved in their binding and the binding site. Drug binding properties of HSA and competitive binding with the most widespread dietary phenolic compounds are also covered.

## 2. Plasma Proteins

Plasma contains various proteins with different functions including the transport throughout the circulatory system of endogenous and exogenous molecules. The binding of small molecular weight drug molecules with plasma proteins is mostly associated with HSA and AAG and, to a lesser extent, globulins and lipoproteins [26].

HSA is a 66 kDa non-glycosylated monomeric protein of 585 amino acids present in the human body at a concentration of 0.53–0.75 mM [20,27]. HSA constitutes ~4.5% of the weight of human blood and helps to maintain osmotic pressure and pH in the blood stream [26,27]. Its principal functions are to transport fatty acids, hormones, drugs, nutrients and inorganic ions and to buffer pH [28,29]. Due to a large content of ionic residues, HSA is highly soluble in water and its flexibility allows specific binding to a wide array of molecules [26]. The polypeptide chain of HSA forms a heart-shaped conformation with approximate dimensions of 80 × 80 × 30 Å, about 67% consisting of α-helices [11,28]. It contains three homologous *α*-helical domains (I–III), each further divided into two subdomains (A and B) [30]. Among them, subdomains IIA and IIIA are two important binding sites. They are delimited by a hydrophobic surface on one side and a positively charged surface on the other side, displaying well determined cavities to specifically bind neutral and negatively charged compounds.

The globulins (α-globulins, β-globulins, and γ-globulin) are a group of globular water-insoluble proteins [31]. AAG, also known as orosomucoid, is acidic, heavily glycosylated (38 to 48 kDa protein, concentration ~12–31 µM), and comprises a single amino acid chain of 204 residues. An acute phase plasma protein, it is the principal extracellular lipocalin present in blood [32]. Multiple drug-binding sites have been reported for AAG, but one appears to be most important, particularly for basic and neutral drugs [8,27]. It should also be considered that if a compound is available as a racemic mixture in blood/plasma, both HSA and AAG are able to bind preferentially to one stereoisomer [25].

To date, two different approaches to assess drug–protein binding can be distinguished. On one hand, separative methods used to determine directly either the unbound drug or the bound drug concentration by separation of the free ligand from the bound species can be classified as conventional methods (equilibrium dialysis, ultrafiltration and ultracentrifugation), high-performance affinity chromatography and capillary electrophoresis–frontal analysis. On the other hand, non-separative methods have been developed to characterize drug–protein interactions. In this sense, spectroscopic methods (UV–visible, fluorescence, infrared, nuclear magnetic resonance, optical rotatory dispersion, and circular dichroism) based on the perturbation of electronic and spectroscopic energy levels of the ligand or the protein and calorimetric techniques (isothermal titration calorimetry and differential scanning calorimetry) have been extensively used. In the last year, computational measurements have also been developed to characterize the polyphenol–protein interactions [1,24].

## 3. Phenolic Compounds and Plasma Protein Binding

PPB and phenolic compounds have been the subject of numerous recent studies, which have focused primarily on structure–affinity relationships. The main observations are summarized in Table 1.

### 3.1. Flavonoids

**Figure 1:**
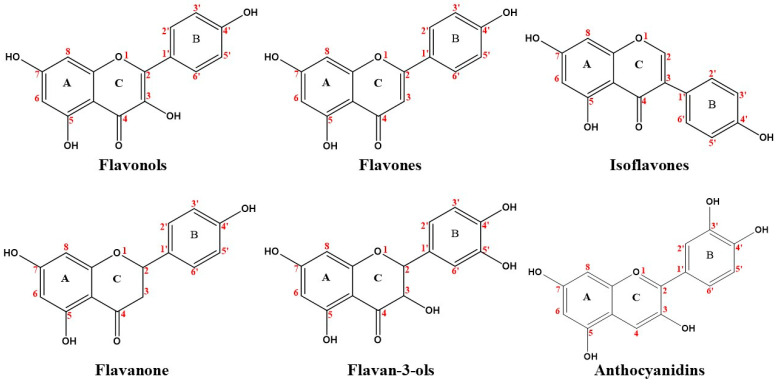
Chemical structure of flavonoids.

Flavonoids are the major polyphenols in a wide variety of plant sources. Associated with antiaging, antifungal, anti-inflammatory, and anticancer activities, they exhibit high reactivity with reactive oxygen species such as hydroxyl, alkoxyl, or peroxyl radicals and efficiently inhibit lipid peroxidation in micelle systems [57]. They have a tricyclic structure in which a phenyl (B ring) is attached at position two or three of a benzopyrone or benzopyran system (AC rings), [1] as shown in Figure 1. The most common sub-classes of flavonoids are flavonols, flavones, isoflavones, flavanones, flavan-3-ols, and anthocyanidins, which are differentiated by the structural features of the C ring [2]. The interactions between flavonoids and plasma proteins have been extensively studied. In 2018, it was found that the position of hydroxyl groups and the presence/absence of an unsaturation between C2–C3 in flavonoids could influence binding with HSA [57].

#### 3.1.1. Flavonols

Flavonols (3-hydroxyflavones) are compounds with medium nucleophilicity and a partial positive charge at the C-3 position [39]. Given that flavonoids are present in the blood circulation mainly as conjugates resulting from phase II metabolic reactions, it is important to know the affinity of flavonoid conjugates for albumin rather than that of their native forms. In this sense, the study of Dufour and Dangles estimated that the majority of flavonol conjugates (more than 80%) circulate bound to albumin rather than in their free form [38]. This transport through the blood circulation is essential for its further delivery at the cell surface, and consequently, membrane uptake to finally produce its biological effect. In this sense, the binding would facilitate the uptake of circulating flavonol conjugates into tissues.

Structurally, the binding affinity of flavonols for HSA is structure-dependent, increasing with the number of OH groups: the addition of a hydroxyl group at C-5 (A ring) increased the binding rate of fisetin with common human plasma proteins (CHPP) by 11.48% [33]. The same occurs for the B ring where the binding constants were in the following order: myricetin > quercetin > kaempferol [34]. Additionally, hydroxylation at C-4’ (B ring) slightly increased the binding affinity of 3,4’-dihydroxyflavone (88.77 ± 0.07%) compared to 3-hydroxyflavone (87.78 ± 0.03%) [35] and reduced that of galangin [33].

Quercetin, one of the most abundant flavonoids in our diet, mainly binds to HSA (subdomain IIA), followed by AAG and with no substantial interaction with very-low-density lipoproteins (99.4 ± 0.1, 39.2 ± 0.5 and <0.5%, respectively) [58]. The binding constants determined for the glucuronide form of quercetin were lower compared to the parent compound, but the stability of quercetin-3-glucuronide and isorhamnetin-3-glucuronide complexes was still high enough to significantly interact with HSA [36]. The main reasons why the glucuronidated form of quercetin had less affinity for HSA are: (1) formation of an intermolecular hydrogen bond or an ionic interaction between the hydroxyl group of quercetin (C-3) and the nitrogen atom of Lys195 [37]; (2) for steric reasons, the presence of the bulky polar substituent (position three) may reduce the PPB affinity of this metabolite; (3) lower complex stabilities due to the absence of interaction of the 3-hydroxyl moiety with Lys195 [36]. On the other hand, the binding experiments reveal that quercetin sulfate lowers its affinity to albumin by one order of magnitude [38]. However, contradictory results were obtained in the study in which the sulfate form of quercetin (quercetin-3’-sulfate) bound to HSA slightly more readily than quercetin [36].

#### 3.1.2. Flavones

Flavones are compounds with medium to high nucleophilicity and a medium partial negative charge at C-3 [39]. Similar to flavonols, these hydrophobic compounds display stronger binding affinities for HSA (higher binding constants) than the other flavonoids [39,59].

Changes in flavone affinity for HSA and CHPP after oxidation or conjugation reactions have been studied. The introduction of a hydroxyl group on the A ring enhanced the HSA binding rate [35]. Another study has found that the optimal number of hydroxyl groups on the A ring of flavone 7-hydroxyflavone, 5,7-dihydroxyflavone (chrysin), and baicalein was one, and the affinities for HSA decreased with the addition of more hydroxyl groups at positions C-5, C-6, and/or C-7 [60]. In 2016, Rimac and coworkers demonstrated that the HSA affinity of flavones was influenced by the location of the OH group, which had an enhancing effect when attached at C-4 on the A ring [39]. Regarding the B ring, the same authors found that after hydroxylation, apigenin had less affinity than chrysin (1.32 ± 0.05 × 10^5^ and 1.95 ± 0.08 × 10^5^ M^−1^, respectively) [39]. Hydroxylation at C-3’ slightly enhanced flavone binding affinity for HSA, which was about 1.38-fold higher in luteolin compared to apigenin [40], a difference that increased to 1.74-fold for CHPP [33]. Hydroxylation of the C ring resulted in a 12.5% higher protein binding rate in 3-hydroxyflavone compared to flavone [35]; hydroxylation at C-3 also increased the affinity of luteolin for CHPP, but little effect was observed in chrysin and apigenin [33].

Hydrogenation of the unsaturated C2=C3 double bond of flavone, 6-hydroxyflavone, and 6-methoxyflavone reduced binding affinities for CHPP by 10.02- to 17.82-fold, but had hardly affect in apigenin and 7-hydroxyflavone [33]. This structural modification had a similar outcome on the affinity of flavone, 6-hydroxyflavone and 7-hydroxyflavone for HSA [35]. The explanation is that the reaction modified the flavonoid C ring from a planar to a twisted structure [33], also reducing polarity, and molecules with a non-planar structure were unable to enter the hydrophobic pockets in HSA [35]. Planarity is therefore an important prerequisite for flavonoid binding in the hydrophobic cavity [39].

Methoxylation increased tangeretin affinity for HSA 100-fold, mainly attributed to enhanced hydrophobicity and hydrophobic interactions [40]. Compared to flavone, the affinity of 6-methoxyflavone was slightly higher, whereas a slightly lower affinity was observed when a hydroxyl group was substituted by a methoxy group (methylation) (6-methoxyflavone < 6-hydroxyflavone) [33]. The same behavior was observed with 5-methoxyflavone and 6-methoxyflavone, whose affinity was reduced by 20% and 4%, respectively. The more methoxy groups were added, the more the protein binding rate decreased (tangeretin > nobiletin) [35].

#### 3.1.3. Isoflavones

Soybean and derived food products are the main sources of dietary isoflavones [1], above all genistein, daidzein and glycitein [61]. Isoflavones have low nucleophilicity and a low partial negative charge [39]. Studies on genistein have indicated it binds to HSA via polypeptide polar groups with overall binding constants of 2.4 ± 0.40 × 10^4^ M^−1^ [62] and 1.5 ± 0.2 × 10^5^ M^−1^ [61]. In the same study, the genistein–HSA complex was found to preclude the attachment of daidzein due to competitive binding. The hydroxylation of isoflavones at positions five and seven on the A ring increased HSA binding affinity rates [35]. This is in contrast with Xiao and coworkers [33], who reported a weakening of isoflavone binding affinities for CHPP after C-5 hydroxylation of the A ring; similar behavior was observed for HSA after C-3’ hydroxylation of the B ring (formononetin > calycosin) [35].

#### 3.1.4. Flavanones

Flavanones have very low nucleophilicity and a high partial negative charge at C-3 on the C ring [39]. In 2011, Cao and coworkers demonstrated that the C2=C3 double bond conjugated with the oxo group at C-4 played an important role in flavanone affinity for plasma proteins; hence, the affinity of apigenin for HSA is about 10,000-fold higher than that of naringenin [40]. Moreover, affinity increased by the addition of a hydroxyl group on the A ring (at C-5 and C-7) and the B ring (at C-2’) [35]. Regarding methoxylation, the addition of a methyl group at position six on the A ring of flavanone slightly increased the protein binding rate [35] and increased the affinity of tangeretin for HSA 100-fold [40] by polarity reduction and capacity improvement to penetrate the tryptophan-rich hydrophobic regions of HSA [40]. Finally, and considering glucuronidation, it showed weakly destabilized flavanone–HSA complexes and the effect was slightly stronger than if it had occurred in the B ring [41].

#### 3.1.5. Flavan-3-Ols

The subclass of the flavonoid family known as flavan-3-ols includes catechins and plant phenols widely distributed in various fruits, red wine, juices, and cocoa, with green tea being the richest dietary source [63]. The number of hydroxyl groups on the B ring and the presence/absence of a galloyl (3,4,5-trihydroxybenzoyl) moiety are responsible for the functional differences in catechins such as (−)-epigallocatechin, (−)-epicatechin, (−)-epicatechin gallate and (−)-epigallocatechin gallate [42,43]. Thus, those with the galloyl moiety showed enhanced hydrophobic properties and consequently higher binding affinities for HSA than catechins lacking this substituent [43,44]. Differences in protein affinity have also been found among stereoisomeric flavan-3-ols [64]. The binding constant (Ka) obtained for (+)-catechin–HSA lies in the intermediate range, not being so low that it leads to an inefficient distribution or so high that it lowers the plasma concentration [42]. The number of binding sites found for the catechin–HSA systems range from 0.87 to 1.10, suggesting that one HSA molecule associates with one catechin molecule [63].

#### 3.1.6. Anthocyanidins

Anthocyanins are water-soluble pigments found throughout plants but are most obvious in fruits and flowers, presenting a spectrum range from pink, red and violet to dark blue [45,46]. The basic chemical structure of anthocyanins is 3,5,7-trihydroxy-2-phenylbenzopyran and they can be classified according to whether they are glycosylated or not, as well as by the number and location of hydroxyl or methoxyl groups and their substituents on the rings [45]. Thus, under physiological pH conditions, the number and position of the hydroxyl groups on the B ring affected anthocyanin affinities for HSA, which increased with the number of groups [45,46,47]. This effect can be explained considering that at pH 7.4 quinoidal and anionic quinoidal bases are major species, which favor hydrogen bonding and other types of electrostatic forces [47]. On the other side, the influence of methoxylation and glycosylation could either strengthen or reduce the anthocyanin affinity for HSA [46,47].

### 3.2. Phenolic Acids

**Figure 2:**
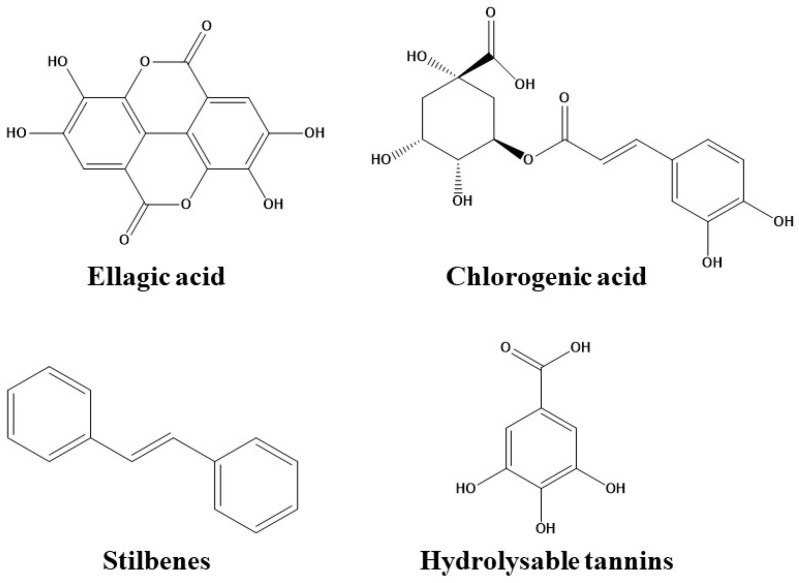
Chemical structure of representative phenolic acids. General chemical structure of stilbenes and hydrolysable tannins.

Phenolic acids, including benzoic and cinnamic acids, are the predominant type of polyphenols in fruits, vegetables, and most plant-derived beverages [49]. Structurally, they derive from benzoic or cinnamic acid, as shown in Figure 2.

#### 3.2.1. Hydroxybenzoic Acids

Ellagic acid, a pharmacologically beneficial polyphenol found in fruits (grapes, strawberries, blackcurrants and raspberries) and nuts [65,66,67], formed a complex with HSA with a binding affinity constant of 15.5 × 10^4^ M^−1^ [65]. In 2014, He and coworkers demonstrated that ellagic acid binds to HSA more strongly than oleuropein [68]. In the case of benzoic acid, the introduction of different numbers and arrangements of hydroxy groups and other substituents on the aromatic ring altered the binding affinities as follows: (1) a hydroxy group at C-2 on the benzene ring exerted a positive effect; (2) a hydroxy substituent at C-4 had a negative influence; and (3) both methylation of the hydroxy groups and substitution of the hydroxy groups with methyl groups at C-3 and C-4 on the benzene ring resulted in an increase [48].

#### 3.2.2. Hydroxycinnamic Acids

The most representative hydroxycinnamic acids are caffeic acid, chlorogenic acid, and caffeoyl quinic acids [1]. The association constants (K) for the HSA–chlorogenic acid interaction at 10, 25 and 40 °C were 6.0 × 10^4^, 9.0 × 10^3^ and 2 × 10^4^ M^−1^, respectively [69], indicating the involvement of two major forces in the binding process. Similar results were reported by Kang and coworkers (4.37 × 10^4^ M^−1^) [50]. The formation of a biomolecular complex between chlorogenic acid and its derivatives with HSA was also studied by Sinisi and colleagues [70]; the reported dissociation constants indicated a very high affinity for this family of compounds, and minimal modifications of the chemical structure led to significant changes in binding. In another study, the binding affinity of chlorogenic acid and its isomers for HSA ranked in the decreasing order: cryptochlorogenic acid > neochlorogenic acid > chlorogenic acid; the 4-esteryl structure was associated with a higher binding affinity and larger conformational changes than the 3- or 5-esteryl structures [51]. The binding constant of HSA complexes was calculated to be 4.29 × 10^4^ M^−1^ at 298 K for rosmarinic acid [49] and 2.23 × 10^4^ M^−1^ for ferulic acid [50]. Cinnamic acid and its hydroxyl derivatives interacted with HSA in the decreasing order of caffeic acid > *p*-coumaric acid > cinnamic acid [52].

### 3.3. Stilbenes

Stilbenoids are an important group of phenolic compounds with a C6-C2-C6 carbon skeleton structure [1], as shown in Figure 2. Among them, resveratrol, found largely in the skin of red grapes and wine, binds to HSA with an association constant of 2.56 × 10^5^ M^−1^ [71]. The effect of hydroxylation of stilbenes has been studied in the context of the structure–affinity relationship of stilbenoid–HSA systems. It has been recently demonstrated that the addition of a hydroxyl group to resveratrol (piceatannol and oxyresveratrol) on the B ring increases the binding rates [35]. In addition, hydroxylation increased the binding affinities of stilbenoids for HSA (pterostilbene > pinostilbene > oxyresveratrol > piceatannol > resveratrol > isorhapontigenin > piceid) [53]. The stilbenoid–HSA affinity was reduced by methylation (resveratrol > pterostilbene) [54] and enhanced by methoxylation at C-3 or C-5 of resveratrol [53].

### 3.4. Hydrolysable Tannins

Little is known about the structural and functional relationship between plasma proteins and tannins. Sekowski and coworkers demonstrated that hydrolysable tannins interact very strongly with HSA, with an intensity of the interaction that depends not only on the number of hydroxyl groups, but also on the bulk, flexibility and hydrophobicity of the chemical structure [55,56]. Tannins with higher molecular weight bind with stronger interaction to proteins, but also tannin flexibility has to be considered: the more flexible the structure, the more rotational capacity of the molecule and easier access to protein binding pockets [55]. Thus, larger and more flexible molecules are capable of changing the secondary structure of albumin through surface interactions, but the presence of valoneoyl structures limit the capacity of tannins to penetrate the hydrophobic tryptophan pocket [55,56].

## 4. Interactions between Phenolic Compounds and Plasma Proteins

### 4.1. Non-Covalent Bonds

Phenolic compounds, under non-oxidative conditions, form reversible complexes with plasma proteins [72,73] involving hydrogen bonds, electrostatic interactions, hydrophobic effects and van der Waals forces [45,72,73]. The interactions between polyphenols and proteins are determined by the thermodynamic parameters of enthalpy (ΔH°) and entropy (ΔS°). Positive values of both parameters indicate a hydrophobic interaction; negative values point to van der Waals forces and hydrogen bonding; and ΔS° > 0 and ΔH° ≈ 0 indicate that an electrostatic force plays a vital role in aqueous solutions [74].

The attraction between the aromatic ring of the polyphenol and hydrophobic regions of other compounds (aliphatic and aromatic side chains of amino acids) leads to the formation of hydrophobic bonds [75]. These bonds arise from changes in entropy rather than enthalpy and are reversible and independent of pH [75]. Hydrogen bonds involve the sharing of a phenolic proton with numerous amide carbonyl moieties of HSA and occur only in the presence of phenoxyl hydrogen. On the other hand, ionic bonds are only generated when polyphenols are ionized to highly reactive phenolate ions. Electrostatic attraction between the phenolate ion and cationic portions of other compounds leads to the formation of this bond [75].

The nature of phenol–protein complexes depends heavily on individual phenolic structures, and the spatial arrangements of substituents seem to be key factors in phenol–HSA affinities [57,72]. In genistein–HSA complexes, hydrophobic and electrostatic interactions play a central role in the binding [76], although the involvement of hydrogen bonds and ionic interactions has also been suggested [61]. The binding between naringin and HSA strongly involves hydrophobic interactions, according to the positive values of ΔH° and ΔS°, but the involvement of electrostatic interactions cannot be ruled out [77]. The nitrogen atom of Lys195 is suitably positioned to establish an intermolecular ionic interaction with the hydroxyl at C-3 in quercetin [37].

To date, numerous studies on flavanone–HSA complex formation have suggested the involvement of: (1) hydrogen bonds between the phenolic hydroxyl groups and the polypeptide chain or polar amino acid residues; (2) hydrophobic interactions between the aromatic ring and hydrophobic amino acid residues; (3) electrostatic forces generated after the deprotonation of the acidic phenolic hydroxyl group in the A ring (hesperetin) by the basic amino acid residues; (4) perpendicular and parallel π−π interactions and (5) van der Waals forces [78,79,80,81].

There is considerable evidence that catechins bind to HSA via hydrophobic and hydrogen bonding [63,64,82]. It has been demonstrated that hydrogen bonding and van der Waals forces are predominant in (+)-catechin–HSA complexes [42]. However, thermodynamic parameters indicate that tea catechins bind serum proteins via ionic interactions (+ΔS° 18 to 9 J mol^−1^ K^−1^ and −ΔH° −13 to −8 kJ mol^−1^) [82].

As anthocyanins are transported in the circulation system, small molecules bound to plasma proteins are exchanged with their free form in order to maintain an equilibrium [29]. Various studies have focused on the structure–molecular affinity relationship of the anthocyanin–HSA interaction. The van der Waals forces, hydrogen bonds, hydrophobic effect and electrostatic forces are the main drivers of the anthocyanin–protein complex formation [29,47]. The strength of anthocyanin binding to HSA is affected by factors such as pH, temperature, and chemical structure. Thus, the hydrophobic effect plays a major role at pH 4 but is less important at pH 7.4. Moreover, at pH 7.4, the association constant of HSA with aglycones bearing an increasing number of hydroxyl groups indicates the importance of attractive electrostatic interactions such as hydrogen bonding between polar groups [47].

Hydrophobic forces and hydrogen bonds are predominant in the HSA binding interactions with phenolic acids and their methyl esters, respectively [48], as well as with ellagic acid [65,66,68]; the involvement of van der Walls forces has also been proposed [67]. Gallic acid is reported to bind to HSA mainly by hydrophobic and electrostatic interaction in microemulsions [83].

Little is known about the structural and functional relationships in tannin–HSA and stilbenoid–HSA complexes. Tannin interaction with plasma proteins was recently described as complex and opportunistic, occurring without specific binding sites and determined mainly by hydrophobic forces and hydrogen bonds [55,56]. On the other hand, the hydrophobic interaction is the main force binding stilbenoids with HSA [53]. Regarding resveratrol, structural analysis showed that it binds non-specifically (H-bonding) via polypeptide polar groups [71].

### 4.2. Hydrolysable Tannins

Highly reactive with oxygen species, many polyphenols can be oxidized to their corresponding semiquinones and quinones, which may then undergo covalent reactions with an enormous number of nucleophiles [73], such as amino acids or thiol groups [72]. In this context, covalent bonds can be formed between a phenolic or quinone carbon ring and plasma proteins. The irreversible polyphenol–protein covalent bindings influence the chemical properties of both species, are resistant to disruption by denaturing agents [73,84,85], and can be formed enzymatically and non-enzymatically [75]. Regarding phenolics, covalent bonds are the result of their electrophilic substitution at the ortho and para positions. On the other hand, conjugate addition reactions to the unsaturated carbonyl moiety lead to the formation of covalent bonds with quinones. The differences in the location and type of substituents are a result of differences in electron densities and distribution both in phenols and quinones. The phenolic polymerization through covalent bonds by oxidative processes (generating phenoxyl radicals), or by condensation reactions between polyfunctional nucleophiles (including the anions derived from polyphenols) and quinones can also be formed [75]. Covalent reactions can change the protein structure with a corresponding modification of hydrophobic–hydrophilic properties of the protein derivatives, accompanied by a change in solubility [73].

In 2005, Kaldas and coworkers demonstrated in an in vitro study that quercetin oxidized by peroxidase/hydrogen peroxide covalently links to proteins, mainly with a high affinity for HSA [84]. However, in 2010, Cahyana and Gordon reported that non-covalent binding occurred between HSA and anthocyanins with a quite weak negative value of Gibbs free energy (ΔG°, −28.2 to −36.3 kJ mol^−1^), which is much lower than the value for covalent bond formation (approximately 400 kJ mol^−1^) [47].

## 5. Binding Sites

HSA contains three homologous *α*-helical domains (I–III), each further divided into two subdomains (A and B) [30]. Subdomains IIA and IIIA (Sudlow’s sites I and II, respectively) are primary binding sites at which many drugs can bind, with IIA being the most prominent. In this sense, bulky heterocyclic anions with the charge located in a central position of the molecules (such as warfarin) are generally attached at the I site of HSA, while aromatic carboxylic acids with an extended conformation and the negative charge located at one end of the molecule (such as ibuprofen) bind at site II [11,38,79,85].

The IIA subdomain appears to be spacious and is comprised of several individual binding sites that can accommodate ligands with a great variety of chemical structures. In contrast, the IIIA site is smaller and less flexible and can only accept structurally similar ligands [39]. The protein microenvironment of site I of HSA is rich in polar (basic) amino acid residues, which are able to stabilize the negatively charged ligand bound in non-planar conformation [37].

In 2005, Dufour and Dangles proposed that the binding of flavonols mainly takes place in subdomain IIA. Computational mapping of possible binding sites of quercetin revealed binding in the large hydrophobic cavity of subdomain IIA [37], similar to that reported for kaempferol [86], hesperetin [79] and apigenin [87].

The higher affinity of flavones for HSA possibly relies on the benzopyrone moiety (AC rings), which protrudes from the hydrophobic cavity and points towards the entrance of site I, as the hydrophilic phenolic hydroxyl groups can interact with nearby polar residues [88]. However, methoxylation decreased flavone polarity and improved the ability to penetrate the tryptophan-rich hydrophobic regions of proteins, which are often buried in the interior of the folded proteins [40]. To date, it is known that the HSA conformation is altered by binding to trimethoxy flavone, with a decrease in *α*-helix content and an increase in β-sheets and random coils, suggestive of the partial unfolding of the secondary structure of the protein [89].

The identification of a binding pocket in HSA for isoflavones suggests daidzein and genistein are bound to the subdomain IIA [61]. Structural differences between curcumin and genistein did not affect the hydrophobic binding affinity for HSA and therefore both are mainly bound in the hydrophobic pockets of Try 214 in site I [62]. Moreover, structural analysis demonstrated that curcumin and genistein bind to HSA via polypeptide polar residues and that the HSA conformation is altered by their complexation, with a reduction in *α*-helix content and an increase in random coil and turn structures, again indicating a partial protein unfolding [62].

Similar to the other flavonoids, the primary binding site for flavanones is in the subdomain IIA, but it is located closer to the binding site in IIIA [40,77,78,80,81]. The alteration of the protein secondary structure has also been studied [77]. When the flavanone concentration was increased, the percentage of protein *α*-helix structure gradually decreased [77,79], which implied a rearrangement of the carbonyl hydrogen bonding network of the main polypeptide chain of the protein [78,79]. The flavanones entered the hydrophobic binding cavity located in subdomain IIA by hydrophobic interactions, and the phenolic hydroxyl groups interacted through strong hydrogen bonds with C=O and N–H of the main polypeptide chain, resulting in the aforementioned rearrangement of the polypeptide carbonyl hydrogen bonding network, and a consequent reduction in the protein *α*-helix structure [79].

A site marker competitive experiment has demonstrated that (+)-catechin is mainly located within site I of HSA [42] and (±), (+), and (−) forms of catechin bind in the proximity of Trp-214 of HSA [64]. Additionally, studies on conformational changes in plasma protein after binding with flavan-3-ols have reported that galloylated catechins increased the electrophoretic mobility of HSA, which reflects a modification of its molecular charge [44,63]. Changes in the α-helicity of the (±), (+), and (−) forms of catechin were found to be marginal [64].

Protein conformational changes can also be attributed to the presence of anthocyanins. Interaction of HSA with keracyanin causes changes in the polarity of the hydrophobic microenvironment. The binding site of keracyanin might be in the hydrophobic cavity, the location of Trp-214 and at the center of the three domains of HSA, which induces slight unfolding or adaptive rearrangement of the polypeptide backbone of the protein [29].

Structural differences affect the binding site of hydroxybenzoic acids. In 2009, a study revealed that protocatechuic acid and its partially methylated products bind to HSA in sites I and II. In contrast, the addition of a hydroxyl group from protocatechuic acid, yielding gallic acid, did not result in competitive binding in sites I or II [90]. An evaluation of the bioactive interaction indicates that the HSA residues for ellagic acid binding are located in subdomain IIA [66] and that the mode of binding reaction is spontaneous [67], which would suggest an alteration of the protein secondary structure. According to circular dichroism spectroscopic data, the fraction of alpha helicity was reduced from 52% to 40% upon binding to ellagic acid [65]. Both ellagic acid and oleuropein interact with the binding pocket of HSA in subdomain IIA [68]. In another study, it has also been demonstrated that gallic acid could bind to the site I of HSA [83].

Differences between hydroxycinnamic acid binding sites have also been studied. Binding with chlorogenic acid induces conformational change in HSA, involving the one tryptophan residue in the binding region [51,69]. The binding of chlorogenic acid and rosmarinic acid most likely takes place in site I and ferulic acid in site II [49,50]. Chlorogenic acids and their derivatives, which are abundant in coffee, form a bimolecular complex within Sudlow’s site I and interact with Trp-214 [70]. Multispectroscopic and docking studies on the binding of chlorogenic acid isomers with HSA show that three isomers bound to HSA at Sudlow’s site I, affecting the protein secondary structure [51]. Chlorogenic acid is thought to bind in subdomain IIA and ferulic acid in IIIA [50]. In another study, it has been demonstrated that after the interaction between HSA with caffeic acid, *p*-coumaric acid and cinnamic acid, the α-helix structure was reduced by 9, 5 and 3%, respectively) [52]. Finally, in 2015, Nair demonstrated through experimental and theoretical studies that both resveratrol and pterostilbene bind to the hydrophobic cavity at site IA in the subdomain II of HSA [54].

## 6. Phenolic Compounds–Drug Interaction

Data on food–drug interactions are generally scarce, despite some well documented exceptions (e.g., grapefruit juice and statins), as food consumption and herbal teas/beverages are not usually monitored in patients. Interactions occur after the concomitant intake of food and drugs, with impacts on the absorption and/or metabolism of the active substance. In some cases, the effects of the interactions may benefit the patients, but they frequently undermine the efficacy of the drug or induce adverse reactions [91].

In the case of PPB, a hormone, drug or even a toxin can be displaced by competing phenolic compounds and then circulates in the blood in a free form. The pharmacodynamics and pharmacokinetics of a drug may subsequently be modified, potentially leading to stronger pharmacological activity, adverse effects and faster elimination [39]. It should be noted that such effects are rarely caused by the formation of phenol–HSA complexes, as the low phenol and high HSA concentration in plasma renders saturation at the binding site unlikely. Moreover, phenolic compounds are often subject to high first-pass metabolism, and thus it is the conjugated-HSA complex that should be taken into account in a potential food–drug interaction. Nevertheless, such an outcome should be kept in mind for drugs with high PPB, a low extraction ratio and narrow therapeutic index, and for other plasma proteins more specific than HSA.

**Figure 3:**
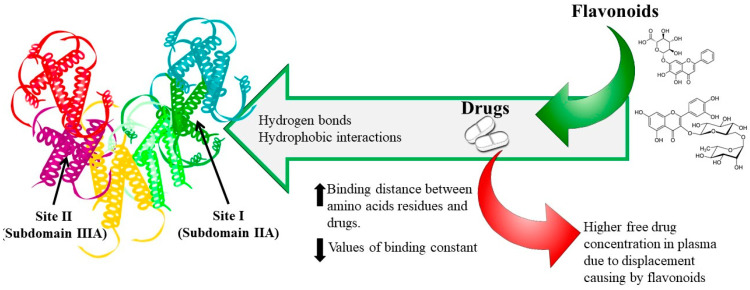
Dietary flavonoid–drug interaction mechanism.

To date, most of the research on food–drug interactions has been focused on flavonoids and HSA. Rutin and baicalin have been extensively used to determine the effect of flavonoids on the binding properties of cleviprex, theophylline, nifedipine, promethazine and ticagrelor [91,92,93,94,95]. The results show that (1) both hydrogen bonds and hydrophobic interactions play a central role in the binding process, which is spontaneous; (2) flavonoids can reduce the association constant and increase the distance of drugs binding to HSA due to competitive binding at site I; (3) the synergistic effect of drugs with rutin and baicalin can further change the HSA conformation, and (4) reduced affinities of drugs binding to HSA in the presence of flavonoids may lead to an increase in free drugs in the blood, which would affect their transportation and/or disposition and may provoke adverse or toxic effects, as shown in Figure 3.

Quercetin had the same effect as rutin and baicalin on ticagrelor and propranolol binding to HSA [95,96]. In 2012, Maciazek-Jurczyk and collaborators reported that competition from curcumin for the binding site of tamoxifen in HSA reduced the binding affinity of this chemopreventive agent, which increased its unbound fraction in the blood with potentially toxic effects [97]. In 2017, Rimac and colleagues showed that warfarin–flavonoid interactions should be regarded as negligible, as they do not share the same binding region in HSA [98]. Conversely, in the same year, it was demonstrated that quercetin metabolites strongly displace warfarin when binding to HSA, suggesting that high quercetin levels can negatively interfere with warfarin therapy [36].

Consequently, the intake of flavonoid-rich foods and beverages should be reduced during treatment with the aforementioned drugs to avoid food–drug interactions and the incidence of toxic symptoms. Alternatively, drugs that do not share the same binding region as flavonoids can be used.

## 7. Conclusions

PPB is a key process that determines the pharmacokinetics (distribution, metabolism, and elimination) and effects of many drugs and dietary phenolics in the body. This review has gathered valuable information concerning the binding of dietary polyphenols to HSA, highlighting how the properties of these compounds can be substantially modified after the formation of a protein complex. The structural differences among flavonoids, phenolic acids, stilbenes, and hydrolysable tannins strongly affect the binding process with plasma proteins. Thus, the formation of a phenolic–HSA complex is affected by phase I reactions. The number and position of substituted hydroxyls on the aromatic ring of the compounds and hydrogenation can alter the nucleophilicity and planarity of molecules. The binding affinities of phenolic compounds towards HSA are also affected by phase II reactions (glucuronidation, methylation, methoxylation and sulfation). Accordingly, in order to avoid potential therapeutic failures caused by food–drug interactions, the intake of flavonoid-rich food and beverages should be monitored when treating certain pathologies.

## Figures and Tables

**Table 1:** Main effect of first pass metabolism of dietary phenolic compounds in human serum albumin (HSA) binding.

Class	Subclass	Reaction	Effect	References
**Flavonoids**	Flavonols	*Hydroxylation*	The binding affinity of flavonols for HSA * is structure-dependent, increasing with the number of OH groups in the A, B ring.	[33,34,35]
		*Glucuronidation*	Decrease the binding constants.	[36,37]
		*Sulfation*	Decrease the binding constants.	[38]
	Flavones	*Hydroxylation*	The HSA affinity of flavones was influenced by the location and number of the OH group.	[33,35,39,40]
		*Hydrogenation*	Hydrogenation of the unsaturated C2=C3 double bond can reduce binding affinities for CHPP **.	
		*Methoxylation*	This reaction enhanced hydrophobicity and hydrophobic interactions increasing affinity for HSA.	[33,35,40]
	Isoflavones	*Hydroxylation*	The hydroxylation at positions 5 and 7 on the A ring increased HSA binding affinity rates. A weakening of isoflavones binding affinities for CHPP after of hydroxylation in C-5 (A ring) and C-3’ (B ring).	[33,35]
	Flavanones	*Hydrogenation*	The C2=C3 double bond conjugated with the oxo group at C-4 plays an important role in flavanone affinity for plasma proteins.	[40]
		*Hydroxylation*	Affinity increased by the addition of a hydroxyl group on the A ring (C-5 and C-7) and the B ring (C-2’).	[35]
		*Methoxylation*	Slightly increased the protein binding rate.	[40]
		*Glucuronidation*	Glucuronidation in the B-ring weakly destabilizes the flavanone-HSA complex.	[41]
	Flavan-3-ols	*Hydroxylation*	The number of hydroxyl groups on the B ring and the presence of a galloyl (3,4,5-trihydroxybenzoyl) moiety increase binding affinities for HSA.	[42,43,44]
	Anthocyanidins	*Hydroxylation*	The binding affinities increase with the number of hydroxyl groups on the B ring.	[45,46,47]
		*Methoxylation*	The methoxylation could either strengthen or reduce the anthocyanin affinity for HSA.	[46,47]
**Phenolic Acids**	Hydroxybenzoic acids	*Hydroxylation*	In the case of benzoic acid, the introduction of (1) an OH group at C-2 on the benzene ring exerted a positive effect and (2) a hydroxy substituent at C-4 had a negative influence.	[48]
		*Methoxylation*	Both methylation of the hydroxy groups and substituting the hydroxy groups with methyl groups at C-3 and C-4 on the benzene ring resulted in an increase of binding affinity.	
	Hydroxycinnamic acids		Minimal modifications of the chemical structure led to significant changes in binding.	[49,50,51,52]
**Stilbenes**		*Hydroxylation*	The stilbenoid–HSA affinity was increased.	[35,53]
		*Methylation*	The stilbenoid–HSA affinity was reduced.	[54]
		*Methoxylation*		[53]
**Hydrolysable Tannins**		*Hydroxylation*	The intensity of the interaction depends not only on the number of OH groups, but also on the bulk, flexibility and hydrophobicity of the chemical structure.	[55,56]

* HSA: human serum albumin; ** CHPP: common human plasma protein.
